# Pollen-mediated gene flow from glyphosate-resistant common waterhemp (*Amaranthus rudis* Sauer): consequences for the dispersal of resistance genes

**DOI:** 10.1038/srep44913

**Published:** 2017-03-22

**Authors:** Debalin Sarangi, Andrew J. Tyre, Eric L. Patterson, Todd A. Gaines, Suat Irmak, Stevan Z. Knezevic, John L. Lindquist, Amit J. Jhala

**Affiliations:** 1 Department of Agronomy and Horticulture, University of Nebraska–Lincoln, Lincoln, NE 68583, USA; 2 School of Natural Resources, University of Nebraska–Lincoln, Lincoln, NE 68583, USA; 3 Department of Bioagricultural Sciences and Pest Management, Colorado State University, Fort Collins, CO 80523, USA; 4 Department of Biological Systems Engineering, University of Nebraska–Lincoln, Lincoln, NE 68583, USA; 5 Northeast Research and Extension Center, Haskell Agricultural Laboratory, University of Nebraska–Lincoln, Concord, NE 68728, USA.

## Abstract

Gene flow is an important component in evolutionary biology; however, the role of gene flow in dispersal of herbicide-resistant alleles among weed populations is poorly understood. Field experiments were conducted at the University of Nebraska-Lincoln to quantify pollen-mediated gene flow (PMGF) from glyphosate-resistant (GR) to -susceptible (GS) common waterhemp using a concentric donor-receptor design. More than 130,000 common waterhemp plants were screened and 26,199 plants were confirmed resistant to glyphosate. Frequency of gene flow from all distances, directions, and years was estimated with a double exponential decay model using Generalized Nonlinear Model (package *gnm*) in R. PMGF declined by 50% at <3 m distance from the pollen source, whereas 90% reduction was found at 88 m (maximum) depending on the direction of the pollen-receptor blocks. Amplification of the target site gene, 5-enolpyruvylshikimate-3-phosphate synthase (*EPSPS*), was identified as the mechanism of glyphosate resistance in parent biotype. The *EPSPS* gene amplification was heritable in common waterhemp and can be transferred via PMGF, and also correlated with glyphosate resistance in pseudo-F_2_ progeny. This is the first report of PMGF in GR common waterhemp and the results are critical in explaining the rapid dispersal of GR common waterhemp in Midwestern United States.

Gene flow refers to both the movement and introduction of genes and gene complexes into ‘allochthonous (distant) gene pools’^1^. Along with gene mutation and natural selection, gene flow is an evolutionary force affecting plant species^2^,^3^. Andrews^4^ demonstrated that natural selection, genetic drift, and gene flow do not work in isolation, and that the presence of one or more of these factors in a population leads to violation of the Hardy-Weinberg assumptions, causing evolution to take place. Spatial restrictions on gene dispersal lead to non-random mating that can result in the transformation of a population-subdivision into genetic neighborhoods, whereas extensive gene flow at the landscape scale promotes homogeneity among plant populations^5^,^6^.

The evolution of herbicide-resistant weeds is a growing threat to modern agriculture and global food security^7^. Gene mutations, initial frequency of resistant alleles, inheritance of resistance genes, fitness, and gene flow are the key factors contributing to the evolution and spread of herbicide-resistant weeds, with the rate of gene flow higher than the rate of mutation in nature^8^. Pollen-mediated gene flow (PMGF) has been occurring since the existence of higher plants and has greatly influenced genetic diversity and the adaptation of populations over time^9^; however, the recent scientific attention to this topic has resulted from concerns regarding transgene movement from genetically modified to non-genetically modified (conventional) crops, as well as their wild relatives^10^,^11^,^12^. Several studies were conducted as part of the risk assessment and environmental biosafety for landscape-level cultivation of herbicide-tolerant crops^13^,^14^; however, limited scientific literature is available on PMGF among different populations of weed species. For example, PMGF has been reported in only a few weed species, including barnyardgrass (*Echinochloa crus-galli* [L.] Beauv.)^15^, common lambsquarters (*Chenopodium album* L.)^16^, kochia (*Kochia scoparia* [L.] Schrad.)^17^, Palmer amaranth (*Amaranthus palmeri* S. Wats.)^18^, rigid ryegrass (*Lolium rigidum* Gaud.)^19^, weedy beet (*Beta vulgaris* L.)^20^, and wild oat (*Avena fatua* L.)^21^.

Common waterhemp (*Amaranthus rudis* Sauer), a summer annual broadleaf weed, is the most commonly encountered weed species in the Midwestern United States^22^. It is a dioecious species belonging to the subgenus *Acnida* (L.) Aellen ex K. R. Robertson of the genus *Amaranthus*^23^. The invasion of common waterhemp in row crop production systems is one of the most drastic events facing agriculture in the Midwest^24^. From the time of its first report in Oklahoma in 1830, common waterhemp has been continuously moving toward the northern and eastern parts of the United States, transforming from a rare wetland weed to a major problem weed^25^,^26^. Over the last several years, common waterhemp has adapted to a wide range of climatic gradients including severe dry conditions^27^, and can now be found from the arid regions of Texas to the humid/sub-humid regions of Maine^28^.

Glyphosate-resistant (GR) common waterhemp was first reported in Missouri^29^, and has now been confirmed in 18 states in the United States^30^, including Nebraska^31^. Its dioecy and anemophilous nature are believed to promote the rapid spread of the herbicide-resistant alleles in an agricultural landscape via pollen migration^32^. Common waterhemp pollen grains are spherical-shaped, polypantoporate (pollen grain with apertures spread over the surface in a regular pattern), and very small in size (18.5 μm diameter), a formation believed to favor their long-distance dispersal^33^,^34^. The rough surface of the pollen grain also creates a boundary layer of turbulence on its exterior, reducing the resistance created when the pollen travels through the air^35^. The lower pollen settling velocity of common waterhemp (0.0185 to 0.021 m s^−1^) compared to other major wind-pollinated crops (e.g., corn, which has a pollen settling velocity of 0.33 m s^−1^ and a pollen diameter of 90 to 100 μm) helps pollen grains to travel longer distances under pollen competition^36^,^37^.

An overproduction of the enzyme EPSPS (5-enolpyruvylshikimate-3-phosphate synthase, the biological target of herbicide glyphosate) through additional *EPSPS* gene copies was responsible for glyphosate resistance in common waterhemp biotypes collected from several Midwestern states^38^,^39^. In a GR common waterhemp biotype from Kansas, Dillon^40^ reported that the amplified *EPSPS* copies were located on a pair of homologous chromosomes, and that the *EPSPS* copies clustered near the centromeres of those chromosomes. *EPSPS* gene amplification was also reported in a GR biotype of Palmer amaranth collected from Georgia, but fluorescence *in situ* hybridization (FISH) analysis suggested that the amplified genes were dispersed throughout the genome^41^.

Because the majority of herbicide-resistant traits are nuclear-inherited and can spread via PMGF^8^, herbicide resistance can be used as an excellent marker to estimate PMGF. The objective of this study was to quantify the frequency of PMGF under field conditions in common waterhemp using glyphosate resistance as a selective marker. This study was conducted using a five-step approach: (i) reviewing literature and constructing hypothesis, (ii) conducting field experiments, (iii) screening for resistance (in greenhouse and laboratory), (iv) fitting statistical models, and (v) predicting gene flow frequency. The null hypothesis for this study was “Gene flow will not occur from glyphosate-resistant to -susceptible (GS) common waterhemp,” which was unrealistic. The hypothesis was constructed to increase the likelihood of detection of the minimum frequency of gene flow and to reduce the type II error.

## Results

### Flowering Synchrony

Flowering was initiated on July 1, 2013 and July 12, 2014, with maximum flowering occurred 2 weeks after flower initiation (Table S1). The common waterhemp plants beyond 5 m distance from the pollen-donor block began flowering a few days later than those in the pollen-donor block or in the proximity of the pollen source. It was believed that the common waterhemp plants in both the pollen-donor block and the closer distances were growing in a higher density compared to the plants at the farthest distances, thus causing a brief gap (<5 days) in the initiation of flowering. The total flowering period lasted about 5 and 4 weeks in 2013 and 2014, respectively. Therefore, a season-flowering synchrony of >85% was ensured (Table S2).

### Meteorological Data

Early and late-season temperature was slightly lower than the temperature during flowering period and mean daily temperature ranged from 17.0 to 30.5 °C in July and early-Aug in 2013 and 2014 (Fig. S1). Pearson correlation coefficients (*r*) showed a significant correlation between frequency of gene flow and wind parameters [wind speed, wind frequency (frequency of time/hours during which the wind blows toward a certain direction), and wind run (calculated by multiplying the average wind speed by the wind frequency for a certain direction^42^)]; however, the correlation (*r* ≥ 0.29) was significant up to 15 m (from the pollen source) for wind speed and 25 m for wind run (Table 1). Correlation between wind parameters and frequency of gene flow at the closer distances (within 5 m) was higher (≥0.48) than at the farthest distances. No negative correlation was observed, suggesting that the wind had a great influence on PMGF in common waterhemp.

**Figure 1:**
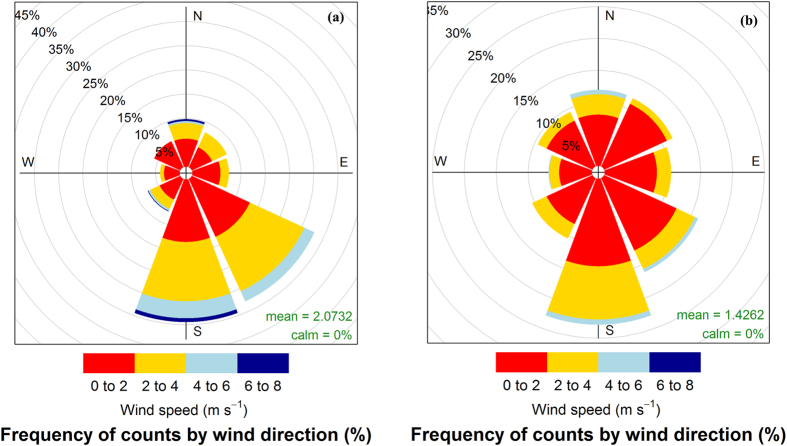
Windrose plots showing the wind speed (m s^**−**1^) and wind frequency (%) in four cardinal (N, S, E, W) and four ordinal (NE, NW, SE, SW) directions at South Central Agricultural Laboratory at the University of Nebraska-Lincoln. During the flowering period in (**a**) 2013 and (**b**) 2014, wind was mostly northbound, and the plots show wind frequency in the directions from which wind was blowing in each year.

Wind flow patterns were similar in 2013 and 2014, though the wind speed and wind frequency differed. Wind during both years blew mostly from the S and SE directions (Fig. 1). The average wind speed during the flowering period in 2014 was lower (1.4 m s^−1^) than the average wind speed in 2013 (2.1 m s^−1^) (Fig. S2).

### Modeling PMGF and Prediction of Distances

Determining the sample size *a priori* allows a researcher to reduce the sampling cost and time without losing statistical precision to quantify gene flow^43^. In 2013 and 2014, more than 130,000 common waterhemp seedlings were screened in the greenhouse to detect PMGF and a minimum power of 0.95 (α = 0.05) was ensured (Tables 2 and 3). Frequency of gene flow was highest at the closest distance (0.1 m) and declined at increasing distances from the pollen source. Averaged across eight directions (N, S, E, W, NE, NW, SE, and SW), gene flow frequency was 0.54 and 0.38 at 0.1 m from the pollen source in 2013 and 2014, respectively (Tables 2 and 3). Mean PMGF frequency was recorded ≤0.10 at a distance ≥35 m.

**Figure 2:**
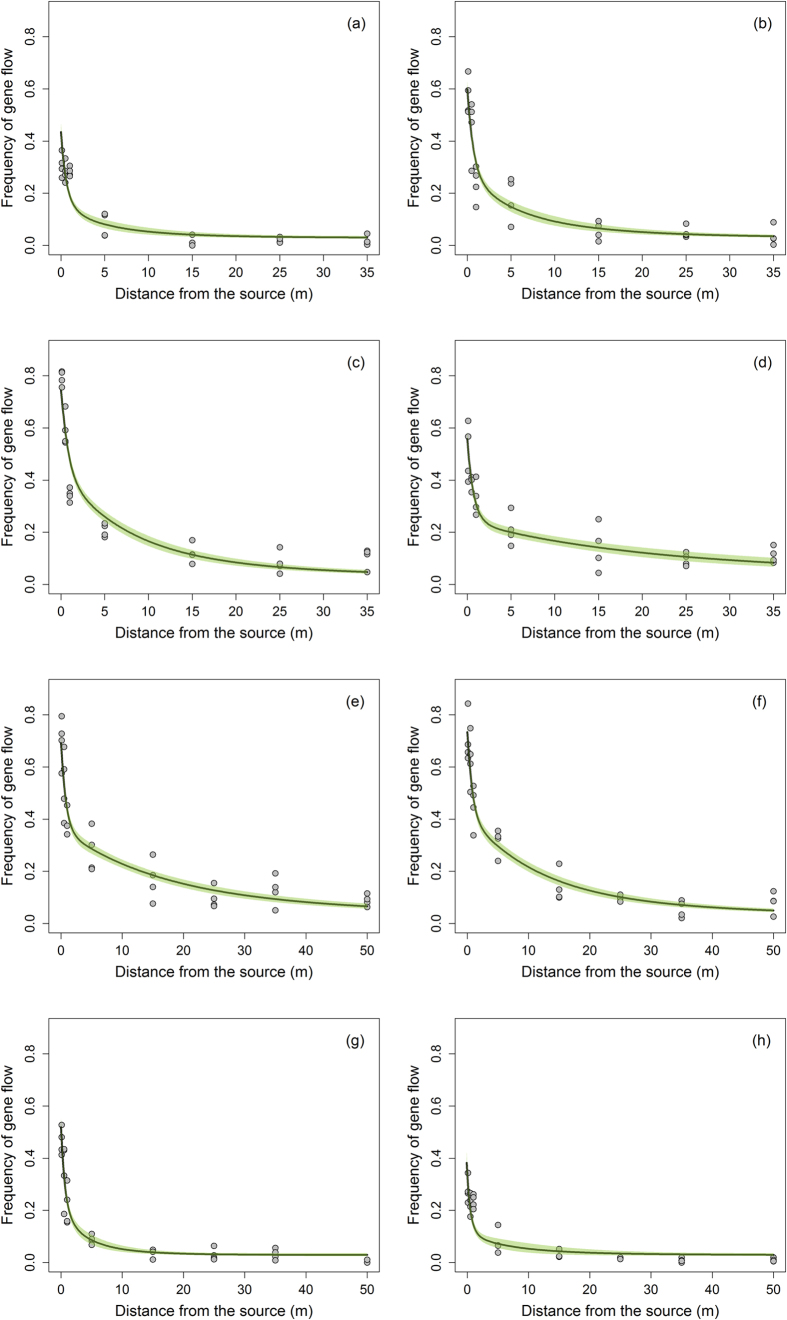
Pollen-mediated gene flow from glyphosate-resistant common waterhemp affected by distance (m) from the pollen source in 2013. Gene flow frequencies from eight directions: (**a**) East, (**b**) West, (**c**) North, (**d**) South, (**e**) Northeast, (**f**) Northwest, (**g**) Southeast, (**h**) Southwest, were plotted against distance and green shaded areas indicate the 95% confidence interval for the prediction plots.

**Figure 3:**
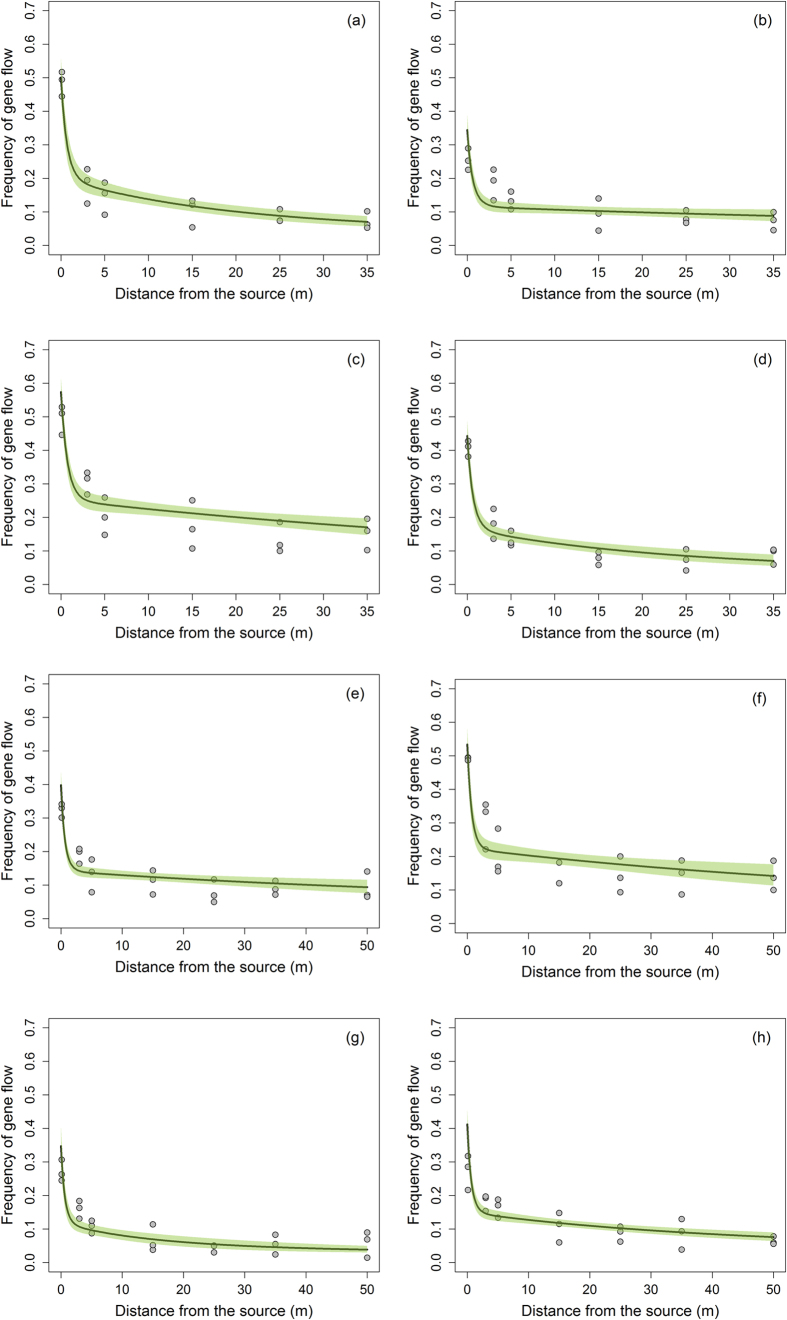
Pollen-mediated gene flow from glyphosate-resistant common waterhemp affected by distance (m) from the pollen source in 2014. Gene flow frequencies from eight directions: (**a**) East, (**b**) West, (**c**) North, (**d**) South, (**e**) Northeast, (**f**) Northwest, (**g**) Southeast, (**h**) Southwest, were plotted against distance and green shaded areas indicate the 95% confidence interval for the prediction plots.

PMGF was not similar in different directional blocks of pollen-receptors or between years. The Akaike’s Information Criterion (AIC)-based model selection procedure [equation (5)] revealed that the inclusion of directions in the final model provided a better estimation for gene flow than the hourly measurements of wind parameters. The ΔAIC (difference in AICs) values between the top model and rest of the models were high (≥398.0), showing the efficacy of the top model for predicting PMGF in common waterhemp (Table S3). Gene flow in common waterhemp declined sharply with increasing distances from the pollen source (Figs 2 and 3), following a double exponential decay model [equation (6)] where the intercepts (β_2_) and decay rates (*γ*_2_) for the second instance varied with different directions and years (see coefficient estimations in Table 4). Pearson’s chi-square test showed a good fit of the model for all directions and years, where the null hypothesis (H_0_, the observed and predicted frequency of gene flow are the same) cannot be rejected due to a P-value ≥ 0.05.

**Figure 4:**
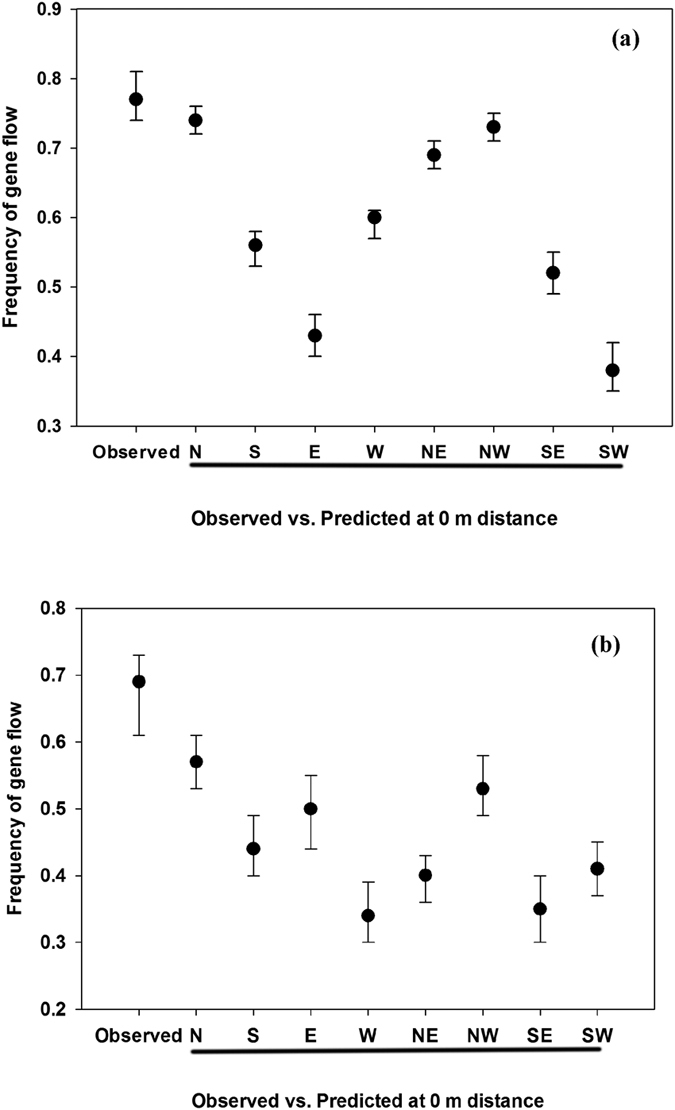
Gene flow frequency at 0 m was predicted using the final model [equation (6)] and compared with the observed frequency at that distance (0 m). Frequency of gene flow (observed and predicted) from (**a**) 2013 and (**b**) 2014 was plotted and the bars above and below each data point indicate a 95% confidence interval.

The gene flow frequencies at 0 m distance were 0.77 and 0.69 in 2013 and 2014, respectively. Gene flow data from 0 m (GS plants planted inside the pollen-donor block) did not include any directions; therefore, could not be included in modeling. However, the final model [equation (6)] has predicted the PMGF frequency at 0 m for each direction and year, which was plotted later along with the original (observed) gene flow frequencies (Fig. 4). In 2013, the model-predicted frequencies (at 0 m) in the N and NW directions were similar to the observed frequency, and in 2014, the gene flow frequency in N direction corresponded with the observed frequency at 0 m.

The predicted distances where gene flow declined by 50% (*O*_*50*_) ranged from 0.6 to 2.4 m in 2013 and from 0.8 to 1.6 m in 2014 (Table 5) depending on the direction being investigated. The maximum distance at which 90% reduction (*O*_*90*_) in the frequency of gene flow was predicted, was 58.3 m in the S direction in 2013 and 87.6 m in the NW direction in 2014. The variability in gene flow increased with increasing distances from the pollen source; therefore, a broad range of *O*_*90*_ values were observed along with wider confidence intervals compared to the *O*_*50*_ values.

### Mechanism and Inheritance of Glyphosate Resistance

**Figure 5:**
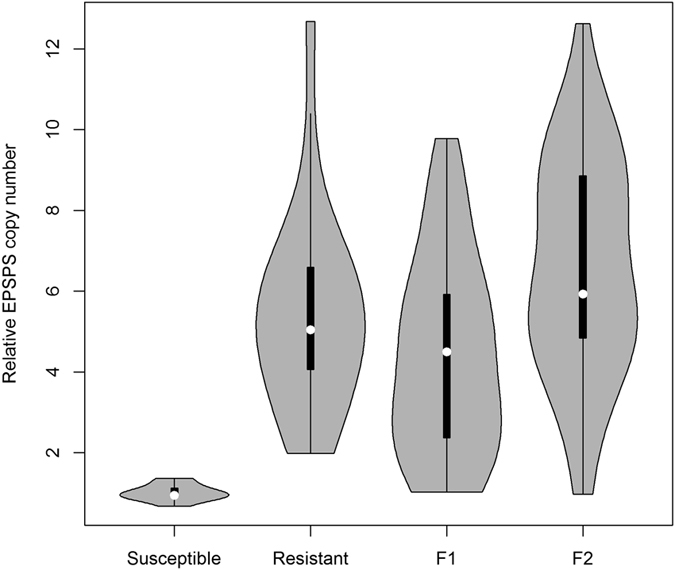
Violin plots combining a boxplot and a kernel density plot to present the distributions of relative *EPSPS* gene copy number in four populations (glyphosate-susceptible and -resistant parents, F_1_, and pseudo-F_2_). The white dot at the center of the boxplot shows the median of the relative *EPSPS* gene copy number.

To determine the mechanism and inheritance of glyphosate resistance, genomic DNA was extracted from young leaf tissues collected from GR and GS parents, F_1_, and an open-pollinated pseudo-F_2_ population. *EPSPS* gene copy number analysis revealed that gene amplification (and the assumed overproduction of EPSPS protein^39^) was the mechanism of glyphosate resistance in the GR common waterhemp parent used in the PMGF study. The mean relative copy number in the GR biotype was 5.3 (±2.2, standard deviation) (Fig. 5). As expected, the GS parent biotype had, on average, one *EPSPS* gene copy (mean = 1.0 ± 0.2). Plants with relative *EPSPS* gene copy number of >1.4 (the threshold copy number) were considered to be GR individuals^44^.

Seeds of F_1_ progeny (GS × GR) were directly taken from the field study (gene flow) and individuals surviving glyphosate application were selected for genomic DNA analysis. Similar to the mechanism of glyphosate resistance in the GR parent, *EPSPS* gene amplification was observed in 92% of the surviving individuals in the F_1_ progeny. For the GR parent, the maximum number of individuals was observed near the median value (5.1) of the relative *EPSPS* copy number, whereas the outcrossing between GS and GR parents resulted in a greater number of plants in F_1_ with a 2.0 to 4.0 *EPSPS* copy number (Fig. 5). *EPSPS* gene amplification was also observed in the pseudo-F_2_ population of common waterhemp with a mean relative *EPSPS* gene copy number of 6.6 ± 2.6 (Fig. 5). It is concluded that the *EPSPS* gene amplification is heritable in common waterhemp and can be transferred via pollen movement under field conditions. The pseudo-F_2_ population of common waterhemp has segregated for both relative *EPSPS* gene copy number and glyphosate resistance; however, the Chi-square test showed that the segregation did not agree with the null hypothesis (Mendelian pattern of segregation for a single gene or locus), which means that F_2_ individuals did not segregate in the 3GR:1GS pattern (Table 6).

### Combining *EPSPS* Gene Amplification with the PMGF from Phenotypic Assessment

**Figure 6:**
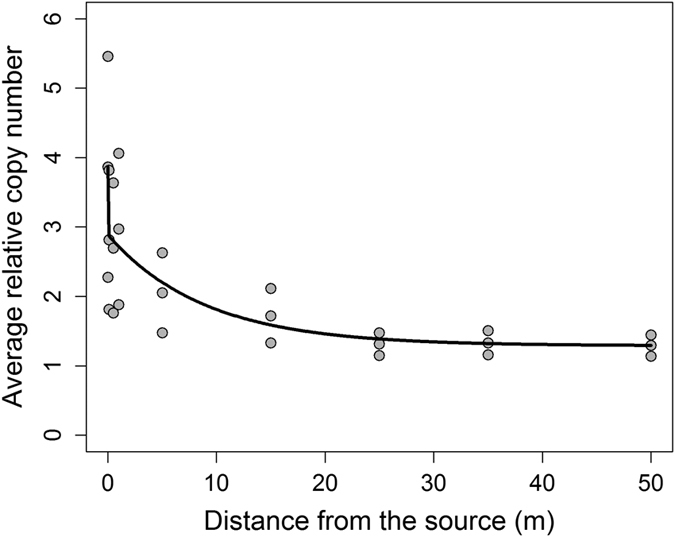
Prediction plot for mean relative *EPSPS* gene copy number in common waterhemp affected by the distance (m) from pollen source.

Mean relative *EPSPS* copy numbers (4.9 ± 2.2) from F_1_ progeny were combined with the gene flow frequency (Tables 2 and 3) to predict an average *EPSPS* gene copy number at any given distance from the pollen source. It was assumed that all GS individuals had a similar *EPSPS* copy number (1.0 ± 0.2). A double exponential decay model was fit to the average copy number data at different distances from the pollen source. Average relative *EPSPS* gene copy number was predicted as 3.9 and 1.9 at the 0 and 10 m distances, respectively, using the equation:


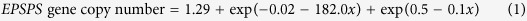


where *x* is the distance from the pollen source. The prediction plot (Fig. 6) for the relative *EPSPS* gene copy number shows the mean relative copy number in the individuals (assumed to be the F_1_ progeny) sampled randomly at a given distance from the pollen source.

## Discussion

The PMGF estimated in this study represents hybridization (or, gene flow frequency) as a function of distance from the pollen source (i.e., actual gene flow), rather than measuring the deposition of pollen from the source to a particular distance (i.e., potential gene flow). Beckie and Hall^45^ noted that it is more relevant to construct a model that predicts the actual gene flow rather than the potential gene flow, especially to understand the transfer of herbicide-resistant traits via pollen movement. PMGF depends on the mating system and reproductive biology of the plant species: for example, in highly outcrossing species such as rigid ryegrass, long-distance (up to 3 km) PMGF has been reported^19^, whereas in self-pollinating species such as barnyardgrass and common lambsquarters, the PMGF was very low (<10%), even at closer distances from the pollen source^15^,^16^. In this study PMGF has been estimated in ideal conditions with no physical or geographic barriers, other vegetation, and no GS male plants in the pollen-receptor blocks, which may have provided more opportunities for gene flow to occur.

The indeterminate growth habit and pulsed flowering pattern of common waterhemp aided in a prolonged flowering period, which ensured a complete flowering synchrony in this study. Wu and Owen^46^ explained that the extended flowering period of common waterhemp is an ecological adaptation to avoid harsh weather conditions and increase the chances of reproductive success. Moreover, long-distance pollen dispersal requires longer pollen viability^47^ and it has been reported that the viability of common waterhemp pollen is about 120 h^32^ with possibility of pollen shed throughout the day^48^,^49^.

Gene flow declined exponentially with distance, which was due to the weaker wind near the leeward edge of the donor block, resulting maximum pollen deposition near the edge of the pollen-donor block. Du *et al*.^50^ suggested that the wind direction near the leeward edge could also be the reverse in some cases. Results of this study also indicated that the average gene flow frequency at 50 m (the farthest distance tested in this study) was ≥0.05 (or ≥5% outcrossing) and the exponential decay model predicted that 90% reduction in gene flow occurred between 10.0 m to 87.6 m from the pollen source, depending on the direction of the pollen-receptor block. Bagavathiannan and Norsworthy^15^ also reported that the average gene flow in barnyardgrass was highly variable among directions and years; therefore, it was important to develop a statistical model that incorporates the effects of direction and year in a single model. A novel statistical approach adopted in this study fulfilled our goal for modeling the PMGF in common waterhemp and can be adopted in future studies to detect PMGF from crops and herbicide-resistant weeds.

The frequency of gene flow never reached 1.00 (i.e., 100% outcrossing) in this study, though the localized GS pollen sources were removed. The presence of GS-F_1_ progeny collected from the pollen-receptor blocks might have occurred for several reasons. Apomictic seed production and the presence of partially monoecious plants have been reported in common waterhemp^51^, and we have observed apomictic seed production in female GS plants grown in the greenhouse, where a few plants produced a small number of seeds without any pollen source and a partially monoecious GS plant was detected during flowering. The GR common waterhemp parent used in this study were derived from a field-collected segregating population and <5% GS male plants were observed in this population; these plants may have contributed pollen to this study, explaining the presence of GS-F_1_ progeny collected from pollen-receptor blocks. Glyphosate resistance by enhanced *EPSPS* copy number seems to be a dominant trait, and therefore susceptible alleles persist in phenotypically resistant plants, which would also result in GS plants in F_1_ progeny. Finally, GS *Amaranthus* species including common waterhemp were present at a distant location from the experimental site, which could have transferred some pollen to the pollen-receptor blocks.

Mallory-Smith *et al*.^52^ outlined the step-by-step approach for conducting a PMGF study under field conditions, and noted that the phenotypic data of herbicide resistance can be considered as an excellent marker, though it must be supplemented with molecular or morphological markers to confirm gene flow. Relative copy number analysis showed that the most GR-F_1_ individuals had increased *EPSPS* gene copy number; however, 4 out of 48 F_1_ individuals surviving glyphosate application possessed a relative *EPSPS* gene copy number of <1.4, which suggested that an additional mechanism could contribute to glyphosate resistance in this biotype. In a common waterhemp biotype collected from Mississippi, Nandula *et al*.^53^ reported both reduced glyphosate translocation and Pro106Ser point mutation in the *EPSPS* gene as potential glyphosate resistance mechanisms. It is possible that the parent GR population used in this study included a small proportion of individuals carrying additional, unknown mechanism(s) of resistance that might have been inherited in the F_1_ progeny, though all the GR individuals selected randomly for genomic DNA analysis showed an increase (>2.0) in *EPSPS* gene copy number. As reported in Palmer amaranth^41^, the results from this study suggested that *EPSPS* gene amplification is also heritable in common waterhemp, though inheritance of *EPSPS* gene amplification did not follow the 3:1 segregation ratio in pseudo-F_2_ progeny. This can be explained by detecting the location of resistant allele(s) on the genome, which was not performed in this study. Recombination within the block of tandem duplication may cause GS individuals to become less frequent than expected, as a large number of individuals would inherit extra gene copies and therefore be GR. If the extra gene copies are at different locations around the genome, the probability of an F_2_ individual inheriting the susceptible genotype (only 1 copy) is less than 25%, which would result in fewer GS individuals than expected.

Estimation of *EPSPS* gene amplification along with the prediction of gene flow provided strong evidence for the pollen-mediated transfer of the glyphosate resistant trait in common waterhemp. Here, the assessment of elevated *EPSPS* copy number was used not only to determine the mechanism of glyphosate resistance but also to confirm inheritance in subsequent progenies. However, *EPSPS* gene amplification could also be used as a molecular marker (instead of growing plants and applying herbicides for screening herbicide resistance) to detect gene flow in other important weed species (such as Italian ryegrass [*Lolium multiflorum* Lam.], kochia Palmer amaranth, ripgut brome [*Bromus diandrus* Roth.], etc.) where increased *EPSPS* copy number has been reported to confer glyphosate resistance^54^.

Results of this study indicated that the glyphosate-resistant trait in common waterhemp is highly mobile and its dispersal is possible via pollen movement depending on the distance from the resistant plants, the wind speed, and the wind direction. Therefore, the mobility of resistant alleles across farm enterprises should be addressed for effective management of this problem weed. Management strategies widely adapted by growers are mostly focused on preventing or delaying resistance evolution over a small area rather than preventing the large-scale movement of herbicide-resistance traits^55^. Field scouting in summer is essential for identifying patches of escaped common waterhemp plants and their management is necessary to prevent PMGF and the spread of resistant alleles. Before pollination, it is essential to identify the male and female common waterhemp plants, so that the removal of female common waterhemp plants, whether mechanically or physically, will reduce the chances of both PMGF and the spreading of herbicide resistance via seed dispersal.

This is the first report describing PMGF from glyphosate-resistant to -susceptible common waterhemp under field conditions. The results of this study addressed the rapid spread of GR common waterhemp in the Midwestern United States and encourage the re-evaluation of management strategies for this troublesome weed species. Being a small-seeded broadleaf weed species, seed-mediated gene flow could play an important role in the dispersal of herbicide-resistant alleles in common waterhemp, as its seeds can also be dispersed by wind, water streams, or by the movement of tillage or harvesting implements. Contaminated crop seeds and manure can also play an important role in seed dispersal. Thus, future research should evaluate the potential for seed-mediated gene flow in common waterhemp. Modeling approach considered in this study to predict the PMGF can also be used for the risk assessment of various transgenic traits in different commercial crops.

## Methods

### Plant Materials

The known GR and GS common waterhemp biotypes were collected from two eastern Nebraska counties (Lancaster and Clay County, respectively); and their sensitivity to glyphosate has been evaluated. The level of glyphosate resistance was estimated 32-fold in the GR common waterhemp biotype compared to the GS biotype^31^.

Seeds from GR and GS biotypes were germinated in the greenhouse using the procedure described by Sarangi *et al*.^31^. The seedlings were transplanted at the cotyledon stage into 72-celled plastic germination trays containing potting mix (Berger BM1 All-Purpose Mix, Berger Peat Moss Ltd., Saint-Modeste, Quebec, Canada) and one seedling per cell was maintained. Plants were kept in the greenhouse and maintained at a constant 28/24 °C day/night temperature, and supplemental light was provided by metal halide lamps at an intensity of 600 μmol photon m^−2^ s^−1^ to maintain a 16 hour photoperiod. Plants were supplied with adequate water and nutrients (Miracle-Gro Water Soluble All Purpose Plant Food, Scotts Miracle-Gro Products Inc., 14111 Scottslawn Road, Marysville, OH 43041). Common waterhemp plants were then transplanted into the field at 8 to 10 cm height.

### Field Experiments

Field experiments were conducted in 2013 and 2014 at South Central Agricultural Laboratory (40.58°N, 98.14°W) at the University of Nebraska-Lincoln. The soil texture at the experimental site was Crete silt loam (montmorillonitic, mesic, Pachic Argiustolls) consisting of 17% sand, 58% silt, 25% clay, 3% organic matter, and a pH of 6.5. The experimental field was under a center-pivot irrigation system and was irrigated when needed. Based on the soil test report, starter fertilizers were applied following the University of Nebraska-Lincoln’s recommendations for soybean^56^. Previous observations listed common lambsquarters, common waterhemp, green foxtail (*Setaria viridis* (L.) Beauv.), Palmer amaranth, and velvetleaf (*Abutilon theophrasti* Medicus) as the primary weed species present at the experimental site; however, there was no report or suspicion of glyphosate resistance in any of these weed species. Field preparations were begun in mid-May by implementing tillage using a tandem disk harrow followed by the application of micro-encapsulated acetochlor [Warrant^®^, 359 g active ingredient (ai) L^−1^, Monsanto Company, 800 N. Lindbergh Blvd., St. Louis, MO 63167] tank-mixed with glyphosate [Roundup PowerMax^®^, 540 g acid equivalent (ae) L^−1^, Monsanto Company, 800 N. Lindbergh Blvd., St. Louis, MO 63167] to control the early-season emergence of weeds, specifically *Amaranthus* sp.^57^. The experimental area and its surroundings (up to 50 m) were either hand-weeded or cultivated later in the season to keep the area free of any *Amaranthus* species or other weeds. The experiments were conducted under a non-crop situation and there were no physical barriers to obstruct gene flow.

**Figure 7:**
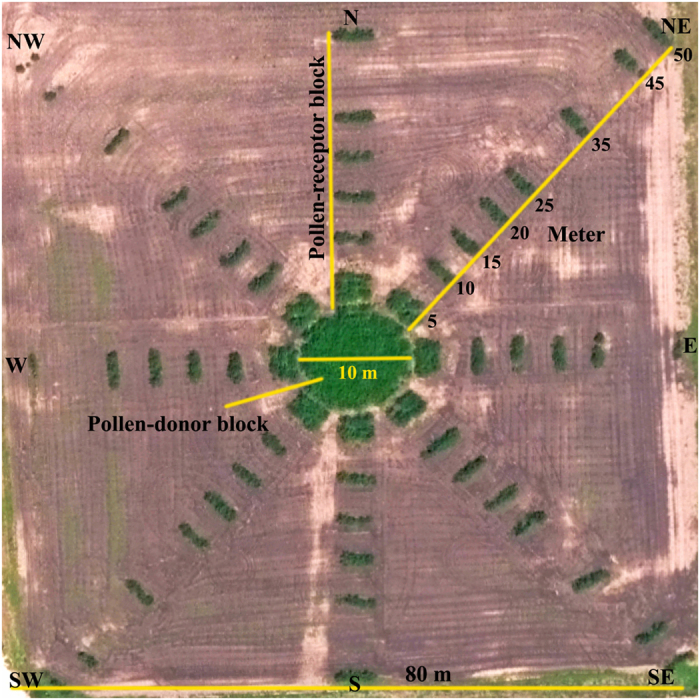
Aerial view of the field experiment conducted to quantify pollen-mediated gene flow from glyphosate-resistant to -susceptible common waterhemp at South Central Agricultural Laboratory at the University of Nebraska-Lincoln. Glyphosate-resistant common waterhemp plants were transplanted in the pollen-donor block of 10 m diameter in the center of the field. The surrounding pollen-receptor area (80 m × 80 m) was divided into eight directional blocks where glyphosate-susceptible common waterhemp plants were transplanted. Common waterhemp seeds were harvested at maturity from specific distances along the eight directional arms. Aerial image is courtesy of Dr. Richard Ferguson, University of Nebraska-Lincoln.

Field experiments were conducted using a concentric donor-receptor design (i.e., a Nelder wheel) where the pollen-donors were surrounded by the pollen-receptors [as described by Jhala *et al*.^43^]. The common waterhemp biotype resistant to glyphosate served as the pollen-donor in this study and the GS plants served as the pollen-receptors. The experimental area was 80 m × 80 m, which also comprised a center circle (80 square m; 10 m diameter) for the pollen-donor block (Fig. 7). Approximately 550 GR common waterhemp plants were transplanted to the pollen-donor block in East-West and North-South directions in a crisscross pattern with a 0.3 m plant-to-plant distance. The transplanting was performed on the first week of June to simulate the typical growing period of common waterhemp in the Midwest for maximum growth and seed production under field conditions^46^.

The receptor area was divided into eight directional blocks (cardinal: N, S, E, and W; ordinal: NE, NW, SE, and SW) and twelve GS common waterhemp plants were transplanted at each of the thirteen specific distances (0.1, 0.5, 1, 3, 4, 5, 10, 15, 20, 25, 35 m for all cardinal and ordinal directions; and additional 45, 50 m only for the ordinal directions) from the pollen-donor block (Fig. 7). Twenty GS plants were also transplanted inside the pollen-donor area (considered as 0 m from the source) to simulate the worst-case scenario of GS plants surrounded by a dense population of GR common waterhemp. GS plants inside the pollen-donor area were marked carefully with flags and plastic tags for easy identification.

### Flowering Period and Seed Harvesting

Male GS common waterhemp plants were visually detected prior to pollen shedding and removed from the pollen-receptor blocks to reduce pollen competition within the receptor blocks. Liu *et al*.^32^ reported that common waterhemp plants can frequently be pollinated by the localized pollen source, which may reduce the chances for long-distance pollen movement. The number of flowering plants (male plants from the pollen-donor and females from the pollen-receptor blocks) was recorded at 5 d intervals and the flowering synchrony was assessed for each direction using the equation:


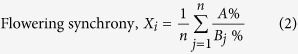


where *n* is the total number of distances in direction *i (i* = N, S, E, W, NE, NW, SE, or SW), *A*% is the percentage of plants shedding pollen in the pollen-donor area, and *B*_*j*_% is the percentage of female flowering plants at the *j*^*th*^ observation (distance) in the pollen-receptor blocks at that specific time. When *X* = 1.0, that means perfect synchrony occurs between pollen-donor and -receptor. *X* > 1.0 shows that sufficient pollen from GR male plants was present to pollinate GS females, but *X* values as low as 0.5 were not considered a good synchrony. Season-flowering synchrony (%) was assessed as the number of days pollen-donor and -receptor flowered together (*X* > 0.8) divided by the total number of flowering days by pollen-receptor female plants, multiplied by 100. Any GS female plants that were late (after 90% of the pollen-donors have completed pollen shedding) in flowering were removed from the receptor blocks.

At maturity, the seedheads of four GS common waterhemp plants from each distance and direction were harvested separately and labeled. Seeds were collected when ≥75% seeds in a seedhead turned into dark-violet or black. The seeds were then cleaned thoroughly and stored separately in airtight polythene bags at 4 °C for 2 months to overcome their limited seed dormancy.

### Meteorological Data

Hourly surface meteorological data were recorded by the Bowen ratio energy balance systems (BREBS) stations of the Nebraska Water and Energy Flux Measurement, Modeling, and Research Network (NEBFLUX) available at the South Central Agricultural Laboratory, Clay Center, NE^58^. Wind frequency, wind speed, and wind run data were used for modeling PMGF in common waterhemp. Other meteorological data such as temperature, humidity, and precipitation were also recorded, as these factors can affect pollen viability and dispersal^59^.

### Resistance Screening

A power analysis reported by Jhala *et al*.^43^ using binomial probabilities was performed to determine the minimum sample size required to accept an outcome without losing the precision of the statistical tests (see SI materials and methods). Seeds collected from individual common waterhemp plants were germinated separately in the greenhouse and evaluated for glyphosate resistance. Plastic trays (51 cm × 38 cm × 10 cm) containing potting mix were used for growing the plants. A maximum of 200 plants were allowed per tray to ensure sufficient glyphosate coverage on the leaf surface. The putative hybrid plants were sprayed at an 8–10 cm height with 1.5× the recommended rate of glyphosate (Touchdown HiTech^®^, Syngenta Crop Protection, LLC, P.O. Box 18300, Greensboro, NC 27419-8300), where 1× = 1,050 g ae ha^−1^. The resistance screening was performed at the 1.5 × rate (1,575 g ae ha^−1^) of glyphosate to obtain more consistency in the response of common waterhemp plants, to ensure the complete control of any GS plant present, and to assure the survival of any GR plant (as the ED_50_ value, the effective dose to control 50% plant population, for GR parent plants was higher than the 1.5× rate of glyphosate; Fig. S3). The number of seedlings surviving glyphosate treatment was recorded at 21 days after herbicide application and the frequency of gene flow at each distance/direction was calculated using the equation:


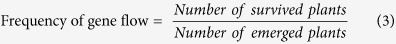


### Modeling PMGF

A novel statistical procedure was used in this study for modeling the PMGF. Rapid reduction in PMGF with increasing distances from the pollen source can be explained by the leptokurtic curve (high probability distribution in the tail) rather than by normal distribution^52^,^60^. The exponential decay, or the exponential power functions, are widely reported statistical models that describe PMGF better^45^.

In this study, all statistical analyses were carried out in R Version 3.2.2^61^. Frequency of gene flow data from all distances and directions and from both years were subjected to nonlinear regression using Generalized Nonlinear Models (package *gnm*). The frequency of gene flow data followed a binomial distribution, as there were two possible outcomes (dead or alive seedlings) from the resistance screening procedure. Unlike the Gaussian distribution, both mean and variance are dependent on the underlying probability, *p*_*i*_, in a binomial distribution. Compared to the nonlinear least square (*nls*) function used in several studies (e.g., Pluess *et al*.^62^), *gnm* has two notable advantages for modeling the PMGF: (i) responses with non-Gaussian distribution can be fitted, and (ii) it is a more convenient way to represent a model with a large number of parameters by symbolic model specification^63^.

In binomial distribution, the probability of success (*p*_*i*_) is a function of the covariate *x* (here, *x* is the distance from the pollen source) that can take any real value, but the *p*_*i*_ should range between 0 and 1 (0 ≤ *p*_*i*_ ≤ 1). Therefore, it was important to transform the frequency of gene flow data to remove the range and floor restrictions; the *Logit*, or log-odds, link function serves this purpose^64^:


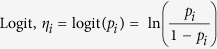



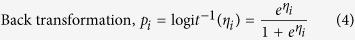


The frequency of gene flow data from both years were described using the exponential decay function in which the independent variables were distance from the pollen source, direction of the pollen-receptor blocks, average wind speed, wind frequency, wind run, and year. Therefore, 62 possible models were constructed and subjected to the test for best fit (Table S3).

### Model Selection

The Akaike’s Information Criterion (AIC) was used for the model comparison and selection of the best model using the equation^65^:


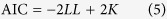


where *LL* is the log-likelihood function for the models and *K* is the number of parameters estimated. The model with the lowest AIC value was considered as the “top model” or best candidate model tested^66^.

### Top Model

A double exponential decay model [equation (6)] where the frequency of gene flow varied with the distance from the pollen source, the direction of the pollen-receptor blocks, and the year provided the best fit to the data (Table S3):


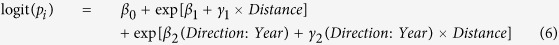


where *p*_*i*_ is the frequency of gene flow of the *i*^*th*^ observation; *β*_0_ is the overall intercept; *β*_1_, and *β*_2_ are the intercepts for the first and second instances, respectively; and *γ*_1_, and *γ*_2_ are the decay rates where *γ*_1_ > *γ*_2_. Here, *β*_2_ and *γ*_2_ vary with the direction and the year.

The distances where the frequency of gene flow was reduced by 50% (*O*_*50*_) and 90% (*O*_*90*_) of the predicted frequency at 0 m were estimated from the final model [equation (6)].

### Model Goodness of Fit

The difference between observed and fitted values for the final model was measured. Model goodness of fit was estimated by Pearson’s chi-squared statistic using the equation^67^:


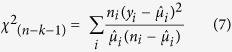


where the sum of the squared difference between *y*_*i*_ (observed values) and 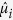 (fitted values for the *i*^*th*^ group of observations) was divided by the variance of *y*_*i*_ that was *μ*_*i*_ (*n*_*i*_ − *μ*_*i*_)/*n*_*i*_ (with *μ*_*i*_ estimated using 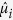) and *n*_*i*_ is the sample size for *i*^*th*^ group. The degree of freedom for Pearson’s chi-squared statistic was *n* − *k* *−* 1, where *n* was the total number of groups and *k* was the number of parameters.

### Mechanism and Inheritance of Glyphosate Resistance

Common waterhemp individuals that survived the resistance screening procedure were considered as hybrids (GS × GR; i.e., F_1_ progeny). Thirty healthy individuals from the F_1_ progeny were selected and transplanted into round plastic pots (20 cm diameter and 30 cm height) containing a 3:1 mixture of soil:potting mix and grown in the greenhouses at the University of Nebraska-Lincoln. Transplanted individuals were again sprayed with glyphosate at 790 g ae ha^−1^ (0.75× the recommended rate) to confirm resistance. Low-level plant injury (chlorosis, stunting, frequent branching, etc.) was observed in less than 50% of the transplanted F_1_ individuals. No mortality was observed, indicating that no susceptible individuals were present (ED_90_ for the GS biotype was 659 g ae ha^−1^; Fig. S3). Ten F_1_ individuals were selected based on their similarities in morphology and flowering initiation time and kept for open pollination in the greenhouse. Seeds were collected at maturity and designated as pseudo-F_2_, as the dioecious nature of common waterhemp prohibits self-pollination of F_1_ plants to form true-F_2_ progeny.

Seeds from the parent biotypes (GR and GS) and the open-pollinated pseudo-F_2_ were germinated in 72-celled germination trays containing potting mix. Emerged seedlings were transplanted at 2 cm height into square plastic pots (10 × 10 × 12 cm). A 50 mg sample of young meristematic leaf tissue was collected from each individual (25 individuals each from the GR and GS biotypes, and 44 individuals from the F_2_ progeny) and were immediately frozen in liquid nitrogen. Individuals were then treated with glyphosate (at the rate of 1,575 g ae ha^−1^; 1.5× the recommended rate) to compare the genomic *EPSPS* copy number data with the greenhouse visual control. Leaf tissue samples were also collected from 48 individuals (3 randomly selected distances × 8 directions × 2 years from gene flow study) of the F_1_ progeny that survived the resistance screening process.

### Sample Preparation

DNA extraction and analysis were performed in the Molecular Weed Science Lab at Colorado State University. Leaf tissue samples were ground to a fine powder using a Qiagen TissueLyser II (Qiagen Inc., Valencia, CA 91355) and a metal bead for 1 min at 30 oscillations per second. Genomic DNA extraction was performed on the leaf tissue using the modified cetyltrimethylammonium bromide (CTAB) method described by Doyle^68^. The concentration and purity of the DNA was measured using a spectrophotometer (Nano Drop 2000 Spectrophotometer, Thermo Fisher Scientific, Wilmington, DE 19810) and each sample was diluted to 10 ng μL^−1^.

### *EPSPS* Genomic Copy Number

Samples were tested for *EPSPS* gene copy number relative to a one-copy reference gene, *CPS* (which encodes the large subunit of carbamoylphosphate synthase [EC 6.3.5.5] and is not associated with any known herbicide resistance)^69^. An 81 base pair (bp) fragment of the *EPSPS* gene was amplified using the forward primer EPSPS_FP1 (5′-GGTTGTGGTGGTCTGTTTCC-3′) and the reverse primer EPSPS_RP1 (5′-CATCGCTGTTCCTGCATTTC-3′). The *CPS* forward and reverse primers were as follows to amplify a 78 bp fragment: CPS_FP1 (5′-ATTGATGCTGCCGAGGATAG-3′) and CPS_RP1 (5′-GATGCCTCCCTTAGGTTGTTC-3′).

For both *CPS* and *EPSPS*, genomic DNA templates (10 ng) were amplified in a 25-μL reaction volume containing forward and reverse primers (400 nM final concentration) and Quanta 2X PerfeCTa qPCR SYBR Green FastMix (Quantabio, Beverly, MA), using a real-time qPCR thermal cycler (Bio-Rad) protocol. The qPCR reactions were heated to 95 °C for 30 s, followed by 40 cycles of 30 s at 95 °C and 30 s at 60 °C. Real-time data for fluorescence of the asymmetrical cyanine dye SYBR Green were recorded at the end of the amplification step in every cycle. A melt curve analysis of the products was performed by heating the PCR products from 60 to 95 °C, in 0.5 °C increments for 5 seconds and reading fluorescence at each step. Melt curve profiles were used to verify a single melting point, indicative of a single PCR product and lack of non-specific products. As a secondary check of amplification specificity, a 5 μL aliquot of the PCR product was run on 1% agarose gel to verify the presence of a single band (Fig. S4).

Relative quantification was conducted using the comparative cycle threshold (*C*_*T*_; 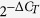) methods described by Schmittgen and Livak^70^. Relative quantification of *EPSPS* for each sample was calculated using the equation:


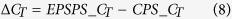


Relative *EPSPS* copy number was expressed as 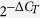. Each individual sample was run in triplicate to calculate the average increase in *EPSPS* copy number and standard deviation.

## Additional Information

**How to cite this article:** Sarangi, D. *et al*. Pollen-mediated gene flow from glyphosate-resistant common waterhemp (*Amaranthus rudis* Sauer): consequences for the dispersal of resistance genes. *Sci. Rep.* **7**, 44913; doi: 10.1038/srep44913 (2017).

**Publisher's note:** Springer Nature remains neutral with regard to jurisdictional claims in published maps and institutional affiliations.

## Supplementary Material

Supplementary Information

## Figures and Tables

**Table 1:** Pearson correlation coefficients# (*r*) between wind parameters (wind speed, wind frequency, and wind run) and frequency of gene flow at different distances†.

Wind parameters		Overall gene flow	Distance from the pollen source‡						
			0.1	1	5	15	25	35	50
			m						
Wind speed	*r* (Pearson)	0.29**	0.65**	0.58**	0.48**	0.29*	0.22	0.19	0.11
	*df*	394	54	30	54	54	54	54	26
Wind frequency	*r* (Pearson)	0.26**	0.57**	0.57**	0.52**	0.42**	0.46**	0.34**	0.40*
	*df*	394	54	30	54	54	54	54	26
Wind run	*r* (Pearson)	0.29**	0.65**	0.56**	0.51**	0.36**	0.35**	0.26	0.15
	*df*	394	54	30	54	54	54	54	26

^#^Pearson correlation coefficients were tested at two significance levels, P < 0.05 (*), and P < 0.01 (**).

^†^Abbreviations: *df*, degrees of freedom.

^‡^Gene flow data at 50 m was only from the four ordinal directions (NE, NW, SE, SW).

**Table 2:** Frequency of pollen-mediated gene flow in common waterhemp from a field experiment conducted at South Central Agricultural Laboratory at the University of Nebraska-Lincoln in 2013.

Distance from pollen source	Plants screened*	Plants with glyphosate-resistant trait	Pollen-mediated gene flow frequency†	Power‡, (1- β); α = 0.05
m	#	#		
0.1	10,281	5,562	0.54	>0.95
0.5	8,115	3,506	0.43	>0.95
1	6,711	3,349	0.50	>0.95
5	5,390	2,032	0.38	>0.95
15	5,987	1,509	0.25	>0.95
25	10,006	630	0.06	>0.95
35	10,498	661	0.06	>0.95
50	10,968	526	0.05	>0.95
Total	67,956	17,775		

^*^Total number of common waterhemp plants screened from all (eight) directions.

^†^Average pollen-mediated gene flow from all (eight) directions.

^‡^Value of power was calculated for a 95% confidence interval using binomial probabilities [equation (S2)].

**Table 3:** Frequency of pollen-mediated gene flow in common waterhemp from a field experiment conducted at South Central Agricultural Laboratory at the University of Nebraska-Lincoln in 2014.

Distance from pollen source	Plants screened*	Plants with glyphosate-resistant trait	Pollen-mediated gene flow frequency†	Power‡, (1- β); α = 0.05
m	#	#		
0.1	5,412	2,030	0.38	>0.95
3	5,634	1,189	0.21	>0.95
5	5,260	805	0.15	>0.95
15	5,687	637	0.11	>0.95
25	10,417	947	0.09	>0.95
35	10,626	1,009	0.10	>0.95
50	20,303	1,807	0.09	>0.95
Total	63,339	8,424		

^*^Total number of plants screened from all (eight) directions.

^†^Average pollen-mediated gene flow frequency from all (eight) directions.

^‡^Value of power was calculated for a 95% confidence interval using binomial probabilities [equation (S2)].

**Table 4:** Estimation of coefficients, standard error, and test of significance for the double-exponential decay model^*^ for prediction of pollen-mediated gene flow from glyphosate-resistant common waterhemp under field conditions.

Coefficients^†^	Estimate	Std. Error	z value	P-value^‡^
*β*_0_	−3.49	0.11	−33.21	<2.0e-16**
*β*_1_	0.32	0.04	8.18	<2.0e-16**
*γ*_1_	−1.25	0.12	−10.55	<2.0e-16**
*β*_2_	0.48	0.10	4.62	3.9e-06**
*γ*_2_	−0.20	0.05	−3.65	<0.001**
*β*_2_: Direction N	0.95	0.10	9.61	<2.0e-16**
*β*_2_: Direction NE	1.13	0.11	10.38	<2.0e-16**
*β*_2_: Direction NW	0.99	0.10	9.65	<2.0e-16**
*β*_2_: Direction S	0.59	0.10	5.70	1.2e-08**
*β*_2_: Direction SE	0.69	0.13	5.42	6.1e-08**
*β*_2_: Direction SW	−0.32	0.13	−2.41	0.02*
*β*_2_: Direction W	0.98	0.12	8.18	<2.0e-16**
*γ*_2_: Direction N	0.10	0.05	1.91	0.06
*γ*_2_: Direction NE	0.15	0.05	2.91	0.004**
*γ*_2_: Direction NW	0.13	0.05	2.55	0.01*
*γ*_2_: Direction S	0.17	0.05	3.24	0.001**
*γ*_2_: Direction SE	−0.03	0.07	−0.51	0.61
*γ*_2_: Direction SW	0.04	0.08	0.51	0.61
*γ*_2_: Direction W	0.06	0.05	1.10	0.27
*β*_2_: Year 2	0.13	0.06	2.08	0.04*
*γ*_2_: Year 2	0.09	0.03	3.24	0.001**
*β*_2_: Direction N:Year 2	−0.41	0.07	−5.66	1.5e-08**
*β*_2_: Direction NE:Year 2	−0.67	0.08	−8.17	<2.0e-16**
*β*_2_: Direction NW:Year 2	−0.46	0.08	−6.02	1.8e-09**
*β*_2_: Direction S:Year 2	−0.35	0.08	−4.37	1.2e-05**
*β*_2_: Direction SE:Year 2	−0.52	0.11	−4.94	7.7e-07**
*β*_2_: Direction SW:Year 2	0.07	0.09	0.79	0.43
*β*_2_: Direction W:Year 2	−0.67	0.10	−6.94	4.0e-12**
*γ*_2_: Direction N:Year 2	−0.04	0.03	−1.57	0.12
*γ*_2_: Direction NE:Year 2	−0.07	0.03	−2.58	0.01*
*γ*_2_: Direction NW:Year 2	−0.06	0.03	−2.20	0.03*
*γ*_2_: Direction S:Year 2	−0.09	0.03	−3.12	0.001**
*γ*_2_: Direction SE:Year 2	0.01	0.03	0.35	0.73
*γ*_2_: Direction SW:Year 2	−0.01	0.04	−34	0.73
*γ*_2_: Direction W:Year 2	−0.02	0.03	−0.76	0.45

*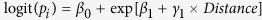 + 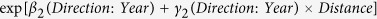, where *p*_*i*_ is frequency of gene flow of the *i*^th^ observation*; β*_0_ is the overall intercept*; β*_1_ and *β*_2_ are the intercepts for the first and second instances, respectively; and *γ*_1_, and *γ*_2_ are the decay rates.

^†^*β*_2_ and *γ*_2_ vary with the direction and the year. In this table, *β*_2_ and *γ*_2_ show the intercept and decay rate, respectively, for one direction (East) in year 1 (2013). However, “*β*_2_:Direction”, or “*β*_2_:Year 2” denote the change (from East direction and year 1) in *β*_2_ for other directions and year 2 (2014), respectively. The same is true for *γ*_2_.

^‡^P-values show the test of significance at P < 0.05 (*) and P < 0.01 (**).

**Table 5:** Estimates of the distances (m) where 50% and 90% reduction in frequency of gene flow occurred in 2013 and 2014*.

Directions	2013				2014			
	*O*_*50*_	*CI*	*O*_*90*_	*CI*	*O*_*50*_	*CI*	*O*_*90*_	*CI*
meter								
N	2.1	1.8; 2.4	22.6	19.5; 26.3	1.6	1.2; 2.4	76.9	52.6; 129.9
S	1.4	1.2; 1.6	58.3	44.4; 76.6	1.1	0.8; 1.4	23.4	16.8; 35.6
E	0.8	0.7; 1.0	13.9	9.3; 19.9	1.2	0.8; 1.7	20.5	15.6; 28.2
W	1.2	1.1; 1.4	16.7	13.1; 21.1	0.9	0.7; 1.3	74.6	39.2; 202.1
NE	2.2	1.8; 2.9	47.8	41.2; 55.7	1.0	0.8; 1.4	74.5	45.6; 153.6
NW	2.4	2.0; 3.0	34.1	29.9; 39.0	1.4	1.0; 2.2	87.6	54.7; 175.0
SE	0.9	0.8; 1.0	10.0	7.3; 13.5	0.9	0.6; 1.3	15.1	9.3; 26.8
SW	0.6	0.5; 0.8	19.9	10.0; 35.2	0.8	0.6; 1.1	33.8	25.7; 48.0

^*^O_*50*_ and *O*_*90*_ are the distances where 50% and 90% reduction in gene flow occurred; *CI* is the 95% confidence interval, which includes the lower and upper values.

**Table 6:** Chi-square* analysis for the segregation in pseudo-F_2_ progeny of common waterhemp based on the relative *EPSPS* gene copy number and phenotypic data.

Progeny	*EPSPS* gene copy number				21 d after glyphosate application†			
	Plants with 1.4 or more copies (GR)	Plants with one copy (GS)	Total	χ^2^	Alive (GR)	Dead (GS)	Total	χ^2^
F_2_	41	2	43	9.49	39	5	44	4.36

^*^The Chi-square goodness of fit test (df = 1, α = 0.05) is used to compare the observed segregation in F_2_ progeny with the expected Mendelian segregation for a single gene (3GR:1GS). The calculated χ^2  ^ > 3.84 indicates that the observed segregation does not agree with the expected segregation.

^†^Glyphosate was applied at 1,575 g ae ha^−1^ and the plant survival data were recorded.
