# Supplementary material for: Pollen-mediated gene flow from glyphosate-resistant common waterhemp (Amaranthus rudis Sauer): consequences for the dispersal of resistance genes
Source: Sci Rep. 2017 Mar 22;7:44913. doi: 10.1038/srep44913 (PMC5361169; doi:10.1038/srep44913)
Supplement: Supplementary Information [file srep44913-s1.doc]

**Manuscript Title: Pollen-mediated gene flow from glyphosate-resistant common waterhemp (*Amaranthus rudis* Sauer): consequences for the dispersal of resistance genes**

Debalin Sarangi^1^, Andrew J. Tyre^2^, Eric L. Patterson^3^, Todd A. Gaines^3^, Suat Irmak^4^, Stevan Z. Knezevic^5^, John L. Lindquist^1^, and Amit J. Jhala^1*^

^1^Department of Agronomy and Horticulture, University of Nebraska–Lincoln, Lincoln, NE 68583; ^2^School of Natural Resources, University of Nebraska–Lincoln, Lincoln, NE 68583; ^3^Department of Bioagricultural Sciences and Pest Management, Colorado State University, Fort Collins, CO 80523; ^4^Department of Biological Systems Engineering, University of Nebraska–Lincoln, Lincoln, NE 68583; ^5^Northeast Research and Extension Center, Haskell Agricultural Laboratory, University of Nebraska–Lincoln, Concord, NE 68728. Correspondence and requests for materials should be addressed to A.J. (email: amit.jhala@unl.edu).

**Supplementary Information**

**Supplementary Methods**

**Dose Response Bioassay.** Greenhouse dose-response bioassays for the parent biotypes (both GR and GS) were conducted and the effective doses of glyphosate needed to provide 90% (ED_90_) and 50% injury (ED_50_) of the parent biotypes were determined using the package *drc* in R^1,2^, using a four-parameter log-logistic model:


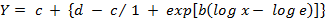
 [S1]

where *Y* is the percent control, *c* is the lower limit, *d* is the upper limit, and *e* represents the ED_50_ values. The parameter *b* is the relative slope around the parameter *e*. The ED_50_ values for the GR and GS biotypes were 1,790 and 263 g ae ha^─1^, respectively, whereas the ED_90_ values were > 16,800 and 659 g ae ha^─1^, respectively (Fig. S3).

**Power Analysis.** A power analysis using binomial probabilities was performed to determine the minimum sample size required to accept an outcome without losing the precision of the statistical tests^3^ using the equation:


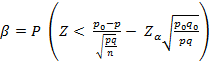


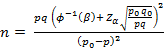


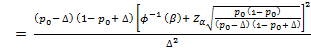
 [S2]

where
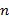
 is the minimum number of seedlings need to be screened;
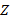
 is the random variable following N (0, 1);
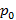
 is the null hypothesized frequency of gene flow;
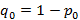
;
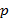
 is the observed frequency of gene flow;
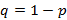
;
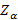
 is the critical value at significant level
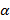
;
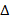
 is the effect size,
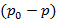
; and
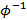
 is the anti-function of the normal curve. Accepting the null hypothesis (H_0_) when it is false is called *Type II error*, whose probability is
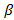
; therefore, the power (1 -
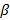
) is the probability of rejecting the H_0_ when it is false^4^.

**Supplementary References**

1. Knezevic, S. Z., Streibig, J. C. & Ritz, C. Utilizing R software package for dose-response studies: the concept and data analysis. *Weed Technol.* **21**, 840–848 (2007).
2. Ritz, C., Baty, F., Streibig, J. C. & Gerhard, D. Dose-response analysis using R. *PLoS One* **10**, 0146021 (2015).
3. Zar, J. H. *Biostatistical Analysis*. 5th edn, 539–542 (Pearson Prentice Hall, 2010).
4. Cohen, J. Statistical power analysis. *Curr. Dir. Psychol. Sci.* 1, 98–101 (1992).

**Supplementary Figures**

Figure S1. Daily average air temperature (°C) from May to October in 2013 and 2014 compared with the 30-year average (1983-2012) at the South Central Agricultural Laboratory at the University of Nebraska-Lincoln. Weather data for the 30-year average were obtained from the High Plains Regional Climate Center (HPRCC: [**http://www.hprcc.unl.edu**](http://www.hprcc.unl.edu/)).

Figure S2. Total number of hours during which wind blew at a particular speed during the flowering period for common waterhemp in the pollen-mediated gene flow experiment conducted at South Central Agricultural Laboratory at the University of Nebraska-Lincoln in 2013 and 2014.


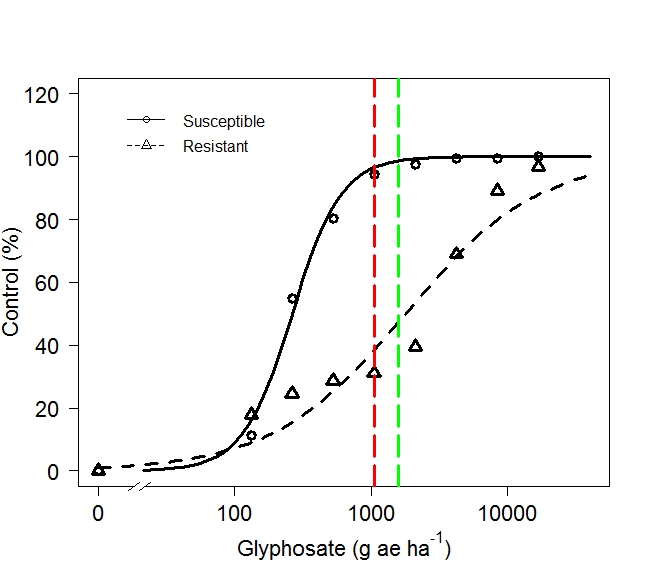


Figure S3. Dose-response bioassay curves for glyphosate-resistant (GR) and -susceptible (GS) parent biotypes used in this study. The weed control estimates were recorded visually at 21 days after glyphosate application. The red dotted line denotes the 1× (= 1,050 g ae ha^─1^) glyphosate rate, while the green dotted line denotes the 1.5× rate. The ED_50_ and ED_90_ values for the GR biotypes were estimated as 1,790 and 21,328 g ae ha^–1^, respectively; whereas the values were 263 and 659 g ae ha^–1^, respectively, for the GS parent biotype.

***EPSPS CPS***

**L S R F_1_ F_2_ S R F_1_ F_2_ L**


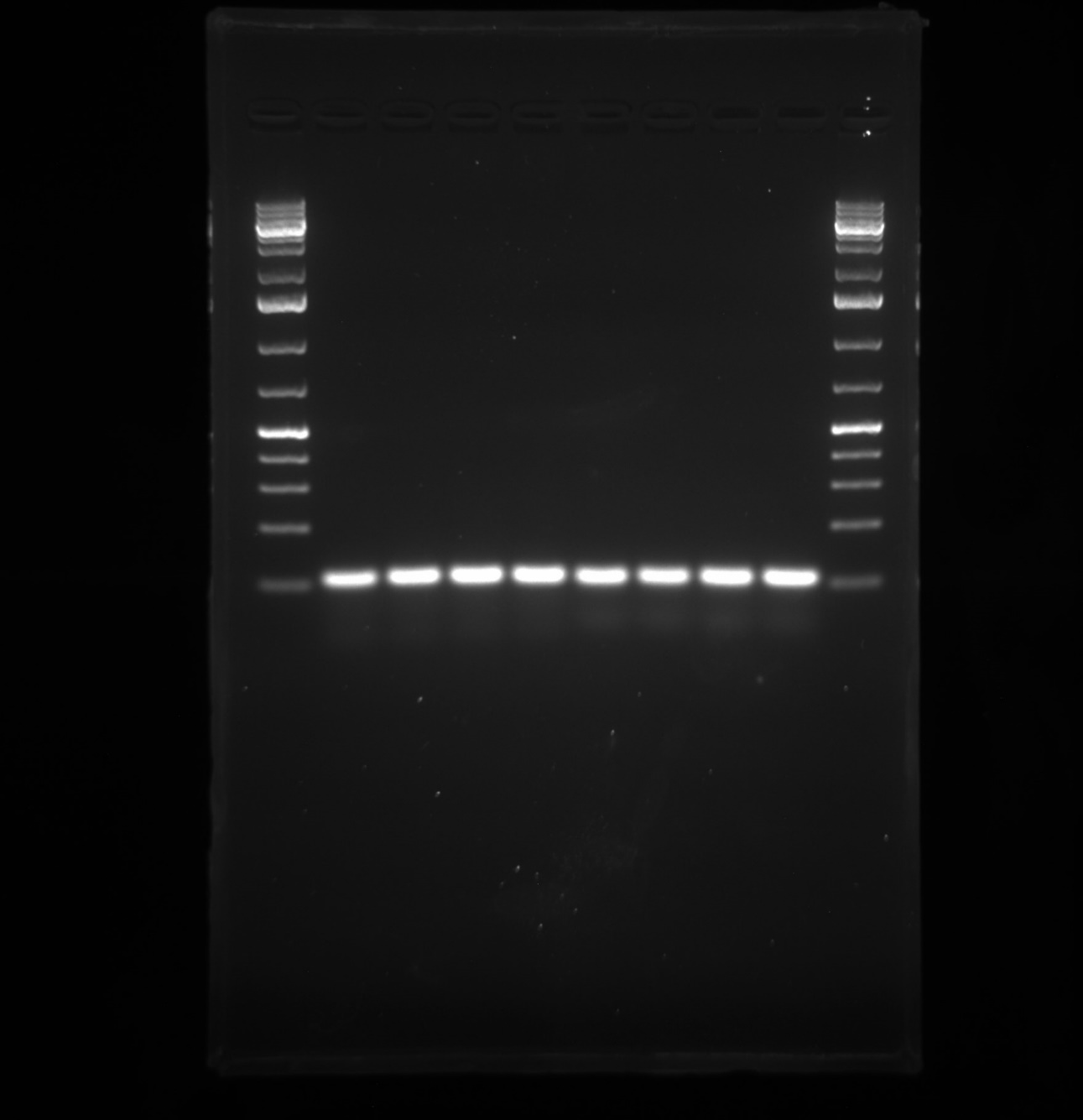


**75 bp-**

**200 bp -**

**Figure S4.** Gel electrophoresis of *EPSPS* and *CPS* PCR products. Single bands correspond to the predicted amplicon lengths for two genes (81 bp, *EPSPS* and 78 bp, *CPS*); L = GeneRuler^TM^ 1 kb Plus DNA ladder, S = glyphosate-susceptible (GS), R = glyphosate-resistant (GR), F_1_ = hybrid between GS × GR, F_2_ = pseudo-F_2_ from the cross of the F_1_ individuals.

**Supplementary Tables**

Table S1. Flowering synchrony between glyphosate-resistant and -susceptible common waterhemp in the pollen-mediated gene flow study conducted in 2013 and 2014.

| Directions | Flowering synchrony^*^ | | | | | | | |
| --- | --- | --- | --- | --- | --- | --- | --- | --- |
|  | 2013 | | | |  | 2014 | | |
|  | July 1 | July 6 | July 16 | July 31 |  | July 12 | July 27 | August 6 |
| N | 1.1 | 1.9 | 1.0 | 1.3 |  | 1.7 | 1.0 | 1.0 |
| S | 1.8 | 2.3 | 1.0 | 1.2 |  | 2.2 | 1.0 | 1.2 |
| E | 0.8 | 0.9 | 1.0 | 1.6 |  | 2.7 | 1.0 | 1.5 |
| W | 1.3 | 1.6 | 1.0 | 1.2 |  | 1.4 | 1.0 | 2.2 |
| NE | 1.8 | 2.2 | 1.0 | 1.2 |  | 2.1 | 1.0 | 0.9 |
| NW | 2.5 | 1.6 | 1.1 | 1.0 |  | 4.5 | 1.0 | 1.3 |
| SE | 1.3 | 1.9 | 1.0 | 1.2 |  | 4.5 | 1.0 | 1.4 |
| SW | 1.1 | 1.1 | 1.1 | 1.2 |  | 2.8 | 1.0 | 1.0 |
| Average | 1.5 | 1.7 | 1.0 | 1.2 |  | 2.7 | 1.0 | 1.3 |
| % flowering plants in pollen-donor block | 25 | 60 | 99 | 50 |  | 35 | 95 | 35 |

^*^ Flowering synchrony was calculated using Equation:
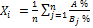
, where
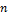
 is the total number of distances in direction *i*,
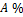
 is the percentage of plants shedding pollen in the pollen-donor area, and
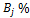
 is the percentage of female flowering plants at the *j^th^* observation (distance) in the pollen-receptor blocks at that specific time.
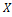
 = 1.0 means perfect synchrony between the pollen donor and the receptor.
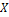
 > 1.0 shows that sufficient pollens from GR male plants were present to pollinate GS females, but
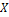
 values as low as 0.5 was not considered a good synchrony.

Table S2. Transplanting, flowering dates, and season-flowering synchrony for common waterhemp in a pollen-mediated gene flow study conducted under field conditions.

| Year | Transplanting dates | Flowering dates | Season-flowering synchrony (%)^*^ |
| --- | --- | --- | --- |
| 2013 | June 5 | July 1 – Aug 5 | 88 |
| 2014 | June 6 | July 12 – Aug 11 | 86 |

^*^ Season-flowering synchrony (%) was assessed as the number of days pollen-donor and -receptor flowered together divided by the total number of flowering days by pollen-receptor female plants, multiplied by 100.

**Table S3. AIC values, and AIC differences (Δ) for the possible models to predict pollen-mediated gene flow (PMGF) under field conditions^^**.

| No. | Models^†^ | *K* | *LL* | *AIC* | *ΔAIC* |
| --- | --- | --- | --- | --- | --- |
| PMGF frequency varying with distance, direction, and year: | | | | | |
| 1 | GF ~ exp(Dist) + exp(Dist*Dir*Yr) | 35 | –1372 | 2813 | 0 |
| 2 | exp(Dist*Dir*Yr) | 33 | –1573 | 3211 | 398 |
| 3 | exp(Dist) + exp(Dist+Dir*Yr) | 20 | –1604 | 3248 | 435 |
| 4 | exp(Dist*Dir) + exp(Dist*Dir) | 33 | –1632 | 3329 | 516 |
| 5 | exp(Dist+Dir*Yr) + exp(Dist) | 20 | –1673 | 3386 | 573 |
| 6 | exp(Dist+Dir) + exp(Dist+Dir) | 19 | –1676 | 3390 | 577 |
| 7 | exp(Dist) + exp(Dist*Dir) | 19 | –1716 | 3470 | 657 |
| 8 | exp(Dist + Dir*Yr) | 18 | –1758 | 3553 | 740 |
| 9 | exp(Dist) + exp(Dist+Dir) | 12 | –1766 | 3555 | 742 |
| 10 | exp(Dist*Dir) + exp(Dist) | 19 | –1868 | 3774 | 961 |
| 11 | exp(Dist*Dir) | 17 | –1881 | 3797 | 984 |
| 12 | exp(Dist+Dir) + exp(Dist) | 12 | –1898 | 3821 | 1008 |
| 13 | exp(Dist) + exp(Dist) | 5 | –2514 | 5038 | 2225 |
| 14 | exp(Dist) | 3 | –2545 | 5096 | 2283 |
| 15‡ | exp(Dist*Dir*Yr) + exp(Dist*Dir*Yr) | – | – | – | – |
| 16‡ | exp(Dist*Dir*Yr) + exp(Dist) | – | – | – | – |
| 17‡ | exp(Dist+Dir*Yr) + exp(Dist+Dir*Yr) | – | – | – | – |
| PMGF frequency varying with distance, wind parameters, and year: | | | | | |
| 18 | GF ~ exp(Dist*WFreq*Yr) + exp(Dist*WFreq*Yr) | 17 | –1780 | 3594 | 781 |
| 19 | exp(Dist*WRun*Yr) + exp(Dist*WRun*Yr) | 17 | –1801 | 3635 | 822 |
| 20 | exp(Dist) + exp(Dist*WFreq*Yr) | 11 | –1852 | 3725 | 912 |
| 21 | exp(Dist) + exp(Dist*WRun*Yr) | 11 | –1874 | 3771 | 958 |
| 22 | exp(Dist*WS*Yr) + exp(Dist*WS*Yr) | 17 | –1906 | 3846 | 1033 |
| 23 | exp(Dist*WFreq*Yr) + exp(Dist) | 11 | –1919 | 3860 | 1047 |
| 24 | exp(Dist*WRun*Yr) + exp(Dist) | 11 | –1932 | 3886 | 1073 |
| 25 | exp(Dist + WRun*Yr) + exp(Dist + WRun*Yr) | 11 | –1943 | 3908 | 1095 |
| 26 | exp(Dist + WS*Yr) + exp(Dist + WS*Yr) | 11 | –1968 | 3958 | 1145 |
| 27 | exp(Dist*WFreq*Yr) | 9 | –1978 | 3975 | 1162 |
| 28 | exp(Dist) + exp(Dist*WS*Yr) | 11 | –1981 | 3984 | 1171 |
| 29 | exp(Dist) + exp(Dist + WFreq*Yr) | 8 | –1985 | 3987 | 1174 |
| 30 | exp(Dist + WFreq*Yr) + exp(Dist) | 8 | –1985 | 3987 | 1174 |
| 31 | exp(Dist*WRun*Yr) | 9 | –1995 | 4008 | 1195 |
| 32 | exp(Dist) + exp(Dist + WRun*Yr) | 8 | –1999 | 4014 | 1201 |
| 33 | exp(Dist + WRun*Yr) + exp(Dist) | 8 | –1999 | 4014 | 1201 |
| 34 | exp(Dist*WFreq) + exp(Dist*WFreq) | 9 | –2010 | 4038 | 1225 |
| 35 | exp(Dist + WRun) + exp(Dist + WRun) | 7 | –2023 | 4059 | 1246 |
| 36 | exp(Dist) + exp(Dist + WRun) | 6 | –2051 | 4114 | 1301 |
| 37 | exp(Dist + WFreq*Yr) | 6 | –2054 | 4120 | 1307 |
| 38 | exp(Dist*WS) + exp(Dist*WS) | 9 | –2053 | 4124 | 1311 |
| 39 | exp(Dist + WRun*Yr) | 6 | –2062 | 4136 | 1323 |
| 40 | exp(Dist*WRun) + exp(Dist) | 7 | –2061 | 4136 | 1323 |
| 41 | exp(Dist) + exp(Dist*WRun) | 7 | –2061 | 4136 | 1323 |
| 42 | exp(Dist + WRun) + exp(Dist) | 6 | –2062 | 4137 | 1324 |
| 43 | exp(Dist + WFreq) + exp(Dist + WFreq) | 7 | –2071 | 4155 | 1342 |
| 44 | exp(Dist*WS*Yr) + exp(Dist) | 11 | –2074 | 4170 | 1357 |
| 45 | exp(Dist + WS) + exp(Dist + WS) | 7 | –2085 | 4184 | 1371 |
| 46 | exp(Dist + WFreq) + exp(Dist) | 6 | –2088 | 4188 | 1375 |
| 47 | exp(Dist*WFreq) + exp(Dist) | 7 | –2088 | 4189 | 1376 |
| 48 | exp(Dist) + exp(Dist*WFreq) | 7 | –2088 | 4189 | 1376 |
| 49 | exp(Dist) + exp(Dist + WS*Yr) | 8 | –2087 | 4190 | 1377 |
| 50 | exp(Dist + WS*Yr) + exp(Dist) | 8 | –2087 | 4190 | 1377 |
| 51 | exp(Dist + WS) + exp(Dist) | 6 | –2096 | 4204 | 1391 |
| 52 | exp(Dist) + exp(Dist + WS) | 6 | –2096 | 4204 | 1391 |
| 53 | exp(Dist) + exp(Dist*WS) | 7 | –2096 | 4206 | 1393 |
| 54 | exp(Dist*WS) + exp(Dist) | 7 | –2098 | 4211 | 1398 |
| 55 | exp(Dist*WRun) | 5 | –2106 | 4222 | 1409 |
| 56 | exp(Dist*WFreq) | 5 | –2117 | 4245 | 1432 |
| 57 | exp(Dist*WS*Yr) | 9 | –2120 | 4257 | 1444 |
| 58 | exp(Dist + WS*Yr) | 6 | –2156 | 4323 | 1510 |
| 59 | exp(Dist*WS) | 5 | –2164 | 4338 | 1525 |
| 60‡ | exp(Dist) + exp(Dist + WFreq) | – | – | – | – |
| 61‡ | exp(Dist + WFreq*Yr) + exp(Dist + WFreq*Yr) | – | – | – | – |
| 62‡ | exp(Dist*WRun) + exp(Dist*WRun) | – | – | – | – |

^*^ *AIC* is the Akaike Information Criterion calculated using Equation 5; *K* is the number of parameters; *LL* is the maximized log likelihood.

^†^ Dist = distance from the pollen source; Dir = directions of the pollen receptor blocks; GF = gene flow frequency; PMGF = pollen-mediated gene flow; WS = wind speed; WFreq = wind frequency; WRun = wind run (i.e., WS × WFreq); Yr = year.

‡ These models failed to converge.
